# Genotype-independent association between vitamin D deficiency and polycystic ovarian syndrome in Lahore, Pakistan

**DOI:** 10.1038/s41598-020-59228-4

**Published:** 2020-02-10

**Authors:** Nasira M. Lone, Saba Riaz, Amna Z. Eusaph, Charles A. Mein, Eva L. Wozniak, Theodoros Xenakis, Zhenqiang Wu, Sidra Younis, David A. Jolliffe, Kashaf Junaid, Adrian R. Martineau

**Affiliations:** 1 Department of Microbiology and Molecular Genetics, University of the Punjab, Quaid-e-Azam Campus, Lahore, 54590 Pakistan; 2 Institute for Population Health Sciences, Barts and The London School of Medicine and Dentistry, Queen Mary University of London, London, E1 2AB UK; 3 Citilab and Research Center, 525-A Maulana Shaukat Ali Road, Block A Faisal Town, Lahore, 54000 Pakistan; 4 Lady Willingdon Hospital, Ravi Road, Walled City, Lahore, 54000 Pakistan; 5 Genome centre, Barts and the London School of Medicine and Dentistry, Queen Mary University of London, London, E12AD UK; 6 School of Population Health, The University of Auckland, Auckland, 1142 New Zealand; 7 Department of Biological Sciences, National University of Medical Sciences, Rawalpindi, 46000 Pakistan; 8 Department of Clinical Laboratory Sciences, College of Applied Medical Sciences, Jouf University, Sakaka, Saudi Arabia

**Keywords:** Genetic association study, Risk factors

## Abstract

Both vitamin D deficiency and single nucleotide polymorphisms (SNPs) in the gene encoding the vitamin D receptor (VDR) have been widely reported to associate with susceptibility to polycystic ovarian syndrome (PCOS). A case-control study was conducted to study the influence of vitamin D status and genotpye for 24 SNPs in four genes in the vitamin D pathway (VDR, DBP, CYP27B1, CYP24A1) on PCOS. Statistical analyses were conducted to identify phenotypic and genotypic factors associated with risk of PCOS and to test for interactions between genotype and vitamin D status. PCOS was independently associated with lower age, higher body mass index, lower waist-hip ratio, vitamin D deficiency (serum 25-hydroxyvitamin D concentration <10 ng/mL), lack of outdoor exercise, increased fasting glucose and a family history of PCOS in at least one first degree relative. No statistically significant association was observed between the genotype of any SNP investigated and risk of PCOS, either as a main effect or in interaction with vitamin D status. We report a strong and independent association between vitamin D deficiency and risk of PCOS in Pakistan, that was not modified by genetic variation in the vitamin D pathway.

## Introduction

Polycystic ovary syndrome (PCOS) is the most common reproductive, endocrine and metabolic disorder in women of reproductive age^1^. Globally, the prevalence of PCOS ranges from 4 to 21%, as defined using NIH 1990 and Rotterdam 2003 criteria^2^. Epidemiological data from Pakistan are scarce, but the prevalence of PCOS has been reported to be particularly high among women of South Asian ethnic origin^3,4^.

PCOS is thought to arise as a result of the interplay between genetic and environmental factors^5^. A growing body of literature indicates that vitamin D deficiency may be a risk factor: meta-analyses of observational studies reveal independent associations between PCOS and low serum concentrations of 25-hydroxyvitamin D (25[OH]D, the major circulating vitamin D metabolite) as well as single nucleotide polymorphisms [SNPs] in the gene encoding the vitamin D receptor (VDR)^6–11^. However, data relating to vitamin D status among women with PCOS in Pakistan are lacking. Moreover, studies in the field have not yet tested for main effects of SNPs in the genes encoding the 1-alpha hydroxylase enzyme CYP27B1 (which converts 25[OH]D to its active metabolite 1,25-dihydroxy vitamin D) or the vitamin D 24-hydroxylase enzyme CYP24A1 (which converts 25[OH]D to its major inactive catabolite 24R,25-dihydroxy vitamin D). Neither have they tested for interactions between SNPs in the vitamin D pathway and vitamin D status in influencing the risk of PCOS.

We have previously demonstrated that genetic variation in the vitamin D pathway can modify the influence of vitamin D deficiency and supplementation on clinical phenotype in the context of tuberculosis^12–14^. We, therefore, conducted a case control study in Lahore, Pakistan, to determine whether vitamin D deficiency and SNPs in four vitamin D pathway genes might influence the risk of PCOS either as main effects or in interaction with each other.

## Results

### Participant characteristics

A total of 235 PCOS cases and 235 healthy controls were recruited to the study between April 2016 and April 2018. Characteristics of cases and controls are presented in Table 1. The mean age of cases vs. controls was 25.2 vs 29.7 years, respectively, and the majority (96.8%) of participants were of Punjabi ethnic origin. Married and single women were equally represented in both groups. Students were over-represented, and employed women were under-represented, in cases vs controls, but the proportion of participants at different educational levels did not differ between groups. As anticipated, acne, hair loss, hirsutism, menstrual cycle abnormalities, and pelvic ultrasound scan abnormalities were all more common among cases vs controls. The mean body mass index was higher among cases vs controls, but the mean waist-to-hip ratio was lower. Mean serum concentrations of fasting glucose and luteinizing hormone were higher in cases vs controls, but no difference was seen for mean serum concentration of the follicle-stimulating hormone. As anticipated LH/FSH ratio was higher in cases vs controls. Mean serum 25(OH)D concentration was lower in cases vs controls (17.4 vs 21.7 ng/ml, respectively; P < 0.001).

**Table 1:** Characteristics of cases and controls.

		Cases (n=235)	Controls (n=235)	Mean difference (95% CI)	Odds ratio (95% CI)	P
Mean age, years (s.d.)		25.2 (4.8)	29.7 (6.8)	4.5 (3.4, 5.6)	—	<0.001
Ethnic origin	Punjabi	222 (94.4%)	233 (99.1%)	—	Ref	
	Others	13 (5.5%)	2 (0.8%)	—	6.82 (1.52, 30.57)	0.01
Marital status	Married	121 (51.5%)	120 (51.1%)	—	Ref	
	Single	114 (48.5%)	115 (48.9%)	—	1.02 (0.71, 1.46)	0.93
Monthly household income, Pakistani Rupees	<25,000	49 (16.6%)	68 (28.9%)	—	Ref	
	25,000-100,000	175 (74.5%)	154 (65.5%)	—	1.57 (1.03, 2.41)	0.04
	>100,000	11 (4.7%)	13 (5.5%)	—	1.18 (0.49, 2.84)	0.72
Occupation	Student	96 (40.8%)	54 (22.9%)	—	Ref	
	Unemployed	108 (45.9%)	112 (47.6%)	—	0.54 (0.35,0.83)	0.005
	Employed	31 (13.2%)	69 (29.4%)	—	0.25 (0.14, 0.43)	<0.001
Education	Illiterate	40 (17%)	34 (14.4%)	—	Ref	
	Intermediate	80 (34%)	89 (37.9%)	—	0.76 (0.44, 1.32)	0.34
	Graduate	115 (48.9%)	112 (47.6%)	—	0.87 (0.51, 1.47)	0.61
Acne	Yes	143 (68.8%)	2 (0.01%)		—	
	No	92 (39.1%)	233 (99.1%)		0.01 (0.00, 0.02)	<0.001
Hairloss	Yes	177 (75.3%)	28 (11.9%)		—	
	No	58 (24.6%)	207 (88.1%)		0.04 (0.03, 0.07)	<0.001
Hirsutism	Yes	193 (82.1%)	0 (0%)		—	
	No	42 (17.9%)	235 (100%)		1	<0.001
Menstrual cycle	Abnormal^a^	210 (89.4%)	0 (0%)		—	
	Normal	25 (10.6%)	235 (100%)	-	1	<0.001
Abnormal pelvic ultrasound	Positive	168 (71.5%)	0 (0%)	—	—	—
	negative	67 (28.5%)	235 (100%)	—	1	<0.001
Mean body mass index, kg/m^2^ (s.d.)		26.7 (5.2)	24.2 (4.3)	−2.4 (−3.3, −1.6)	—	<0.001
Mean waist-to-hip ratio percentage(s.d.)		80.6 (5.8)	82.7 (3.3)	2.2 (1.3,3.0)		<0.001
Mean fasting glucose, mg/dL (s.d.)		103.2 (21.3)	86.1 (7.7)	−17.5 (−20.3, −14.8)	—	<0.001
Mean luteinizing hormone concentration (LH), IU/L (s.d.)		10.9 (8.0)	5.9 (3.0)	−5.0 (−6.1,-3.9)	—	<0.001
Mean follicle-stimulating hormone concentration (FSH), IU/L (s.d.)		5.5 (2.9)	5.4 (2.0)	−0.1 (−0.5, 0.4)	—	0.75
Mean LH/FSH ratio (s.d)		3.1 (5.0)	1.2 (1.0)	−1.9 (−2.5, −1.2)	—	<0.001
Mean serum 25(OH)D, ng/ml (s.d.)		17.4 (16.2)	21.7 (7.9)	4.3 (1.9, 6.6)	—	<0.001

a, Abnormal menstrual cycle in Cases: amenorrhea: [28 (11.9%), menorrhagia: 18 (7.6%), oligomenorrhea: 164 (69.7%)].

### Association between phenotypic features and risk of PCOS

Table 2 presents the results of univariable and multivariable analyses testing for associations between participant characteristics captured by the study questionnaire or found at physical examination and susceptibility to PCOS. PCOS was independently associated with lower age (adjusted odds ratio [aOR] per additional year of age, 0.86; 95% CI 0.81 to 0.93), higher body mass index (aOR per additional kg/m^2^, 1.26; 95% CI 1.15 to 1.37), lower waist-hip ratio (aOR per additional unit, 0.88; 95% CI 0.82 to 0.95), vitamin D deficiency (serum 25(OH)D <25 ng/ml, aOR 24.81; 95% CI 6.16 to 99.84), lack of outdoor exercise (aOR 13.47; 95% CI 6.26 to 28.97), increased fasting glucose (aOR per 1 mg/dL increase, 1.12; 95% CI 1.09 to 1.17) and a family history of PCOS in at least one first degree relative (aOR 2.75; 95% CI 1.12 to 6.78). The following features were not found to associate with susceptibility to PCOS: monthly household income, or self-reported consumption of sugary/fast foods, chicken, eggs, milk or red meat.

**Table 2:** Determinants of PCOS risk from study questionnaire and physical examination.

		N	Proportion of cases	Proportion of controls	Univariable analysis		Multivariable analysis	
					Unadjusted OR (95% CI)	P	Adjusted odds ratio (95% CI)^a^	P
Age, years		470	—	—	0.87 (0.84, 0.90)	<0.001	0.86 (0.81, 0.93)	<0.001
BMI, kg/m^2^		470	—	—	1.12 (1.07, 1.1)	<0.001	1.26 (1.15, 1.37)	<0.001
Waist: hip ratio, %		470	—	—	0.91 (0.86, 0.94)	<0.001	0.88 (0.82, 0.95)	<0.001
Deseasonalized serum 25(OH)D, ng/ml	≥10	381	149/235 (63.4%)	232/235 (98.7%)	—	—	Ref	—
	<10	89	86/235 (36.6%)	3/235 (1.3%)	44.63 (13.86, 143.72)	<0.001	24.8 (6.16, 99.84)	<0.001
Monthly household income, Pakistani Rupees	<25,000	117	49/235 (16.6%)	68/235 (28.9%)	—	—	—	—
	25,000-100,000	329	175/235 (74.5%)	154/235 (65.5%)	1.57 (1.03, 2.41)	0.04	—	—
	>100,000	24	11/235 (4.7%)	13/235 (5.5%)	1.18 (0.49, 2.84)	0.72	—	—
Family history of PCOS^b^	No	393	184/235 (78.3%)	209/235 (88.9%)	—	—	—	—
	Yes	77	51/235 (21.7%)	26/235 (11.0%)	2.23 (1.34, 3.72)	<0.001	2.75 (1.12, 6.78)	0.03
Outdoor exercise	Yes	284	76/235 (32.3%)	208/235 (88.5%)	—	—	—	—
	No	186	159/235 (67.7%)	27/235 (11.5%)	16.12 (9.92, 26.18)	<0.001	13.47 (6.26, 28.97)	<0.001
Fasting glucose, mg/dL		470	—	—	1.11 (1.09, 1.14)	<0.001	1.12 (1.09, 1.17)	<0.001
Sugary / fast food consumption	No	286	129/235 (54.9%)	157/235 (66.8%)	—	—	—	—
	Yes	184	106/235 (45.1%)	78/235 (33.2%)	1.65 (1.14, 2.40)	<0.001	1.13 (0.56, 2.29)	0.72
Chicken consumption	Daily/weekly	96	51/235 (21.7%)	45/235 (19.1%)	—	—	—	—
	Less often	374	184/235 (78.3%)	190/235 (80.9%)	1.20 (0.75, 1.83)	0.49	—	—
Egg consumption	Daily/weekly	347	180/235 (76.6%)	167/235 (71.1%)	—	—	—	—
	Less often	123	55/235 (23.4%)	68/235 (28.9%)	0.75 (0.49, 1.13)	0.17	—	—
Milk consumption	Daily/weekly	268	89/235 (37.9%)	179/235 (76.2%)	—	—	—	—
	Less often	202	146/235 (62.1%)	56/235 (23.8%)	5.24 (3.51, 7.81)	<0.001	1.45 (0.72, 2.92)	0.29
Red meat consumption	Daily/weekly	133	61/235 (26.0%)	72/235 (30.6%)	—	—	—	—
	Less often	337	174/235 (74.0 %)	163/235 (69.4 %)	1.25 (0.84, 1.89)	0.26	—	—

Abbreviations: BMI, body mass index; 25(OH)D 25-hydroxyvitamin D.

a, Adjusted for age, BMI, waist-to-hip ratio, deseasonalized 25(OH)D <10 ng/ml vs ≥10 ng/ml, outdoor exercise, fasting glucose, sugary/fast food consumption and milk consumption.

b, PCOS in at least one first-degree relative.

### Association between vitamin D pathway genotype and risk of polycystic ovarian syndrome

Table 3 presents results of univariable and multivariable analyses testing for associations between SNPs in the genes encoding the vitamin D receptor (VDR), the vitamin D 1-alpha hydroxylase enzyme (CYP27B1), the vitamin D 24-hydroxylase enzyme (CYP24A1) and the vitamin D binding protein (DBP) and risk of PCOS. No statistically significant association was observed between the genotype of any polymorphism investigated and risk of PCOS.

**Table 3:** Genotypic determinants of PCOS risk.

Gene	SNP	Genotype	Proportion with PCOS	Unadjusted OR (95% CI)	P for unadjusted OR	Adjusted odds ratio (95% CI)^a^	P for main effect	P for interaction between genotype and vitamin D status^b^
VDR	rs1544410	CC	143/284 (50.4%)	0.93 (0.68, 1.26)	0.64	0.78 (0.46, 1.33)	0.37	0.99
		TC	77/152 (50.7%)					
		TT	11/26 (42.3%)					
VDR	rs2228570	GG	56/110 (50.9%)	0.97 (0.75, 1.25)	0.75	0.93 (0.59, 1.47)	0.76	0.93
		GA	109/218 (50%)					
		AA	63/127 (49.6%)					
VDR	rs7970314	AA	89/173 (51.5%)	0.99 (0.77, 1.27)	0.92	0.72 (0.46, 1.15)	0.17	0.99
		GA	97/200 (48.5%)					
		GG	46/89 (51.6%)					
VDR	rs4334089	GG	15/31 (48.3%)	1.03 (90.77, 1.39)	0.82	0.92 (0.56, 1.52)	0.74	0.30
		GA	73/148 (49.3%)					
		AA	138/275 (50.2%)					
VDR	rs731236	AA	89/193 (46.1%)	1.20 (0.92, 1.56)	0.17	1.66 (1.02, 2.69)	0.04	0.78
		GA	106/204 (52%)					
		GG	37/68 (54.4%)					
VDR	rs7976091	CC	119/229 (52%)	1.02 (0.78, 1.34)	0.85	0.94 (0.58, 1.53)	0.81	0.98
		TC	78/167 (46.7%)					
		TT	32/55 (58.2%)					
VDR	rs2853559	GG	135/277 (48.7%)	1.15 (0.85, 1.54)	0.36	1.28 (0.71, 2.30)	0.41	0.99
		GA	81/157 (51.6%)					
		AA	17/30 (56.6%)					
VDR	rs7975232	AA	17/33 (52%)	0.78 (0.58, 1.05)	0.10	1.00 (0.57, 1.75)	>0.99	0.92
		CA	90/162 (55.6%)					
		CC	116/255 (45.5%)					
VDR	rs10783219	AA	27/49 (55.1%)	0.97 (90.74, 1.29)	0.85	1.06 (0.65, 1.73)	0.87	0.56
		TA	92/198 (46.4%)					
		TT	101/203 (49.7%)					
VDR	rs2238136	CC	111/222 (50%)	1.06 (0.81, 1.39)	0.64	0.92 (0.56, 1.52)	0.75	0.98
		TC	79/173 (45.6%)					
		TT	32/55 (58.2%)					
VDR	rs4516035	TT	35/68 (51.5%)	1.07 (0.82, 1.39)	0.61	1.08 (0.68, 1.69)	0.74	0.53
		TC	95/203 (46.8%)					
		CC	97/185 (52.4%)					
VDR	rs11568820	CC	25/43 (58.1%)	1.01 (0.77, 1.34)	0.92	1.28 (0.80, 2.15)	0.31	0.69
		TC	68/152 (44.7%)					
		TT	137/266 (51.5%)					
DBP	rs2070741	TT	129/251 (51.4%)	0.92 (0.68, 1.22)	0.56	1.08 (0.65, 1.80)	0.76	0.66
		GT	83/172 (48.3%)					
		GG	17/35 (48.6%)					
DBP	rs7041	AA	46/112 (41.1%)	1.14 (0.88, 1.47)	0.31	1.20 (0.77, 1.86)	0.41	0.054
		CA	128/225 (57%)					
		CC	61/126 (48.4%)					
DBP	rs4588	GG	19/43 (44.2%)	0.96 (0.73, 1.27)	0.77	0.92 (0.57, 1.48)	0.72	0.99
		TG	98/179 (55%)					
		TT	117/241 (49%)					
DBP	rs16846876	AA	152/300 (51%)	0.96 (0.70, 1.33)	0.83	0.74 (0.41, 1.33)	0.32	0.99
		TA	66/139 (47.5%)					
		TT	11/20 (55%)					
DBP	rs2298849	AA	178/356 (50%)	1.11 (0.74, 1.64)	0.61	0.94 (0.45, 1.96)	0.86	0.98
		GA	46/97 (47.4%)					
		GG	6/7 (86%)					
DBP	rs12512631	TT	78/157 (49.6%)	0.97 (0.76, 1.24)	0.82	0.96 (0.62, 1.49)	0.85	0.93
		TC	105/201 (52.2%)					
		CC	49/103 (47.6%)					
CYP24A1	rs6013897	TT	107/213 (50.2%)	1.06 (0.81, 1.39)	0.65	1.42 (0.86, 2.36)	0.17	0.72
		TA	96/194 (49.5%)					
		AA	31/56 (55.4%)					
CYP24A1	rs2762939	GG	101/214 (47.2%)	1.14 (0.88, 1.49)	0.32	1.37 (0.84, 2.25)	0.19	0.63
		GC	104/196 (53.1%)					
		CC	29/56 (52%)					
CYP24A1	rs2248137	CC	94/185 (51%)	0.91 (90.70, 1.18)	0.48	0.93 (0.45, 1.96)	0.39	0.98
		GC	101/201 (50.3%)					
		GG	34/75 (45.3%)					
CYP24A1	rs2762934	GG	40/74 (54.1%)	0.96 (0.75, 1.25)	0.79	0.93 (0.58, 1.49)	0.71	0.82
		GA	97/199 (49.7%)					
		AA	96/189 (51%)					
CYP27B1	rs4646536	AA	1/1 (100%)	0.72 (0.32, 1.60)	0.42	1.70 (0.34, 8.56)	0.52	0.99
		GA	12/22 (54.5%)					
		GG	216/436 (49.5%)					
CYP27B1	rs4646537	TT	105/216 (48.6%)	1.06 (0.80, 1.39)	0.69	0.98 (0.61, 1.56)	0.92	0.98
		GT	97/185 (52.4%)					
		GG	24/49 (48.9%)					

Abbreviations: PCOS, polycystic ovarian syndrome; SNP, single nucleotide polymorphism.

a, Adjusted for age, body mass index, waist-to-hip ratio, deseasonalized 25(OH)D <10 ng/ml vs ≥10 ng/ml, outdoor exercise and fasting glucose. b, P value for interaction between genotype and 25(OH)D <10 ng/ml vs ≥10 ng/ml is presented.

### Association between vitamin D deficiency and host genotype, stratified by vitamin D status

We next proceeded to investigate whether genetic variation in VDR, CYP24A1, CYP27B1 and DBP modified the association between vitamin D deficiency and risk of PCOS reported above. P values for interaction for these sub-group analyses are presented in Table 3: these revealed no evidence to support the hypothesis that polymorphisms in the vitamin D pathway modify the effect of vitamin D deficiency on risk of PCOS.

### Haplotype analysis

We also performed haplotype analysis to assess the cumulative impact of all SNPs on risk of PCOS. The Clark method^15^ was used to define haplotypes for SNPs showing significant linkage disequilibrium (R^2^ > 0.80) in the relevant 1000 genomes reference set ([PJL], Punjabi from Lahore, Pakistan). Two SNPs in *VDR* showed linkage disequilibrium with R^2^ > 0.80: rs11568820 and rs7976091. Univariate regression analysis for haplotypes TT, TC and CC showed no significant association with the odds of PCOS. Further analysis was performed by adjusting these haplotypes for the phenotypic risk factors for PCOS identified above (age, BMI, WHR, vitamin D status, outdoor physical exercise and fasting glucose). No statistically significant associations with risk of PCOS were identified (Table 4).

**Table 4:** Haplotype analysis.

Haplotype	Presence	Proportion with PCOs	Unadjusted OR (95% CI)	p-value of unadjusted OR	Adjusted OR (95% CI)^a^	p-value
CC	No	134/256 (52.3%)	Ref	—	—	
	Yes	90/189 (47.6%)	0.83 (0.57, 1.20)	0.32	0.70 (0.36, 1.38)	0.31
TT	No	117/227 (51.5%)	Ref	—		
	Yes	107/218 (49.1%)	0.91 (0.625, 1.31)	0.60	1.08 (0.56, 2.09)	0.81
TC	No	55/95 (57.9%)	Ref	—		
	Yes	169/350 (48.3%)	0.68 (0.43, 1.07)	0.09	0.66 (0.29, 1.46)	0.34

a, Adjusted for age, body mass index, waist-to-hip ratio, deseasonalized 25(OH)D <10 ng/ml vs ≥10 ng/ml, outdoor exercise and fasting glucose.

## Discussion

We report findings from one of the largest and most detailed studies conducted to date to investigate the influence of vitamin D deficiency and genetic variation in the vitamin D pathway on susceptibility to PCOS – and the first such study to be conducted in Pakistan. Uniquely, in addition to investigating SNPs in VDR and DBP, we also explored the influence of variants in CYP24A1 (the gene encoding the major enzymes of 25(OH)D catabolism) and CYP27B1 (the gene encoding the enzyme responsible for converting 25[OH]D to its active metabolite 1,25[OH]_2_D) on risk of disease. Our major findings were that lower vitamin D status was independently associated with susceptibility to PCOS, but that genetic variation in VDR, DBP, CYP24A1, and CYP27B1 was not. Moreover, we found no evidence of any interaction between serum 25(OH)D levels and genetic variation in the vitamin D pathway on disease risk.

Our finding that vitamin D deficiency associates independently with PCOS risk in women in Pakistan is consistent with reports from other settings including the Netherlands^10^, Iran^16^, Egypt^17^ and the USA^18^, as well as with findings from a meta-analysis including these and other studies^19^, showing that mean 25(OH)D level was lower in women with PCOS than controls. The high prevalence of vitamin D deficiency seen among participants in the current study is in keeping with our previous study in Lahore showing a high prevalence of vitamin D deficiency among women of reproductive age^20^. These and other studies reporting high rates of vitamin D deficiency in other groups^21^, highlight that vitamin D deficiency is a major public health problem in Pakistan^22^. The adjusted odds ratio for the association between vitamin D deficiency at the pre-specified 25(OH)D cut-off of 10 ng/ml was higher than has been reported elsewhere; this primarily reflects the very low prevalence of 25(OH)D values <10 ng/ml among controls in the current study. Exploratory analyses investigating 25(OH)D cut-offs of 15 ng/ml (aOR 3.41, 95% CI 1.77 to 6.57), 20 ng/ml (aOR 1.79, 95% CI 0.96 to 3.37), or analyzing 25(OH)D as a continuous variable (aOR 0.98, 95% CI 0.96 to 1.01) showed consistent inverse associations between 25(OH)D level and PCOS risk. Our findings with regard to other factors associated with PCOS, such as higher BMI, reduced physical exercise, higher fasting glucose levels and positive family history are also in keeping with the literature^22–25^.

In contrast to these positive findings, the lack of association seen between polymorphisms in *VDR* in our study contrasts with findings of a recent meta-analysis reporting that the VDR ApaI (rs7975232) polymorphism associated with susceptibility to PCOS in Asian populations (aOR for allelic model, C vs A, 1.19; 95% CI 1.07 to 1.34)^6^. The lack of association seen for SNPs in DBP is consistent with findings of both the other studies that have investigated polymorphisms in this gene^26,27^.

Our study has several strengths. Cases comprised a broad range of PCOS phenotypes, including obese and lean, fertile and infertile, and those with and without hyper-androgenic characteristics. We collected detailed information on potential confounders of the association between vitamin D status and PCOS risk, minimizing the potential for residual/unmeasured confounding to explain our findings. We also investigated SNPs in a wider range of vitamin D pathway genes than previously investigated, and applied a stringent correction for multiple testing. By deseasonalizing 25(OH)D data, we were able to calculate individuals average 25(OH)D level throughout the year, which represents their vitamin D status more effectively than a season-specific ‘snapshot’ provided by a single unadjusted reading^28^.

Our study also has some limitations. Due to the case-control design, reverse causality and/or confounding cannot be excluded as explanations for the associations observed. Although large by comparison with others in the field, our power to detect modest genetic effects, and gene-environment interactions, was limited. 25(OH)D was measured by ELISA rather than with Liquid Chromatography Tandem Mass Spectrometry, which is the gold standard methodology; however, this should not have introduced bias, since the same assay was used to measure vitamin D status in cases and controls. Moreover, the assay we used detects both 25(OH)D_2_ and 25(OH)D_3_.

## Conclusion

In conclusion, this large case-control study—the first of its kind to be conducted in Pakistan—reports that low vitamin D status associates independently with increased susceptibility to PCOS. However, no statistically significant association between polymorphisms in vitamin D pathway genes and risk of PCOS was demonstrated.

## Methods

### Study design

We conducted a case control study. Cases were patients aged 14 to 49 years diagnosed with PCOS and recruited from out-patient clinics at the Jinnah Hospital and the Lady Willingdon Hospital, Lahore. A total of 235 women were selected according to Rotterdam criteria, 2003^29^. Patients with thyroid and adrenal diseases and androgen-secreting tumours were excluded. 235 healthy controls were selected from the Citi lab and Research centre, Lahore, on the basis of having no history of infertility, no evidence of clinical hyperandrogenism and normal menstrual cycles. Informed consent was taken from all participants who fulfilled eligibility criteria. Written informed consent was taken from the parents or guardians of the participants who were under 18 years of age. Participants completed a detailed questionnaire including details of age, weight, height, sociodemographic status, dietary habits, physical exercise, sun exposure, androgenic features (acne, hirsutism and patchy hair loss), menstrual cycle history, family history of PCOS and fertility details for married participants. The study was approved by the ethical committee of the University of Punjab (Ref No: Bioethic 125Pu, 13.10.17) and by the ethical review board of Citi lab and Research Centre, Lahore (Ref # 26-17/ERB/CLRC/27^th^/Dated 28-07-2016). All the methods were performed following the relevant guidelines and regulations approved by the ethical committee.

5 mL of blood was drawn on the 2^nd^ or 3^rd^ day of menstrual cycle^30^, from a median cubital vein; 2 mL were transferred into vials containing EDTA and frozen at −20 °C for subsequent DNA extraction, and 3 mL was added to serum vials and sent to the laboratory within two hours of collection, where serum was isolated from clotted blood by centrifugation and stored at −20 °C for subsequent determination of 25(OH)D concentration.

### Serum 25(OH)D assay

Serum 25(OH) D concentration was determined by ELISA (Calbiotech, EI Cajon U.S.A). Calibrators and controls for the assay were run in duplicates. Interassay CV for serum 25(OH)D for our samples was 14%. Season-adjusted (deseasonalized) values for 25(OH)D were calculated for each participant from their individual standardized 25(OH)D concentration and date of blood sample collection, using a sinusoidal model with values derived from standardized values for all participants as previously described^28,31^. Vitamin D deficiency was defined using a pre-specified 25(OH)D cut-off of 10 ng/ml; this cut-off was selected a priori on the basis that it is widely used by Public Health bodies^32^ and that deficiency at this level has been shown to associate most strongly with PCOS^11^ and other pathologies attributable to vitamin D deficiency^33–35^.

### Genotyping

Genomic DNA was extracted from whole blood and quantified using a nanodrop spectrophotometer as previously described^36^. DNA TaqMan allelic probe assays (Applied Biosystems, Foster City, CA, USA) were used to genotype polymorphisms in genes encoding: the vitamin D receptor (rs4334089, rs10783219, rs4516035, rs11568820 [cdx2], rs7976091, rs731236 [TaqI], rs2228570 [Fok]I, rs1544410 [BsmI], rs7975232 [ApaI], rs7970314, rs2853559, rs2238136); the 1α-hydroxylase enzyme, CYP27B1 (rs4646536, rs4646537); the 24-hydroxylase enzyme, CYP2A41 (rs6013897, rs2762939, rs2248137, rs2762934), and the vitamin D binding protein, DBP (rs7041, rs4588, rs12512631, rs2070741, rs2298849, rs16846876). Taqman® SNP genotyping assays were purchased directly from Life Technologies. Assay IDs are detailed in Supplementary Table 1. One assay was a custom design and primer details are shown in Supplementary Table 2. For SNP Genotyping using the Fluidigm 192.24 Dynamic Array, genomic DNA concentration was normalised to 50 ng/µL and a sample pre-mix was prepared with 2 µL of GTXpress™ Master Mix (2×), 0.2 µL of 20X Fast GT Sample Loading Reagent, 0.2 µL of nuclease-free water and 1.6 µL of genomic DNA. A 10X SNP assay mix was prepared by combining 1.0 µL of Taqman® SNP Genotyping assay with 2.0 µL of 2X Assay Loading Reagent, 0.2uL of ROX™ (50×) and 1.3 µL of nuclease-free water. 3 µL of sample pre-mix and 3 µL of assay mix into each assay inlet of the 192.24 arrays. Pressure fluid was then pipetted into the appropriate wells. The array was then loaded to the Juno™ controller with the RX insert and the Fast PCR 192.24 script run to performs thermal cycling. Thermal conditions were as follows: Hot start 95 °C for 5 minutes, Touchdown (64 °C-61 °C dropping 1 °C per cycle) 4 cycles at 95 °C for 15 seconds, 64 °C 45 for seconds, 72 °C for15 seconds, 34 cycles were run at 95 °C for 15 seconds (denaturing) followed by 60 °C for 45 seconds (annealing) and 72 °C for 15 seconds (extension). Endpoint fluorescent data were collected using the Biomark® Real-Time PCR System, and data were subsequently analysed using the Fluidigm SNP Genotyping Analysis software.

### Statistical analysis

Statistical analyses were done with Stata IC (version 15.1). Frequencies of alleles and genotypes were compared using chi-square tests; all were found to be in Hardy-Weinberg equilibrium. Baseline characteristics of cases vs controls were compared using unpaired Student’s t-tests and chi-square tests for continuous and categorical variables, respectively. Chi-square tests were used to test for associations between independent variables and risk of PCOS in univariate analysis. Binary logistic regression was used for multivariate analysis of phenotypic determinants of PCOS risk, with adjustment for factors found to associate with PCOS with P < 0.05 in the univariate analysis of phenotypic determinants. Binary logistic regression was also used to test for association between genotype and risk of PCOS, using an additive model and adjusting for phenotypic factors found to associate independently with PCOS risk (age, body mass index, waist-to-hip ratio, deseasonalized 25(OH)D <10 vs. ≥10 ng/ml, outdoor exercise and fasting glucose). Sub-group analyses were performed to determine whether genetic variation in the vitamin D pathway modified effects of vitamin D status on susceptibility to PCOS by repeating primary efficacy analysis with the inclusion of a term for the interaction between vitamin D status and genotype, using an additive model. Haplotype analysis was performed using the Clark method. Odds ratios are presented with 95% confidence intervals and P values. The Benjamini-Hochberg procedure for multiple testing correction^37^ was applied to genetic analyses to control the false discovery rate (FDR) at 5%.

### Power and sample size

Assuming the risk of vitamin D deficiency (serum 25[OH]D <10 ng/ml) in the control arm to be 56%^38^, we calculated that 235 cases and 235 controls would need to be recruited in order to detect an odds ratio for the association between vitamin D deficiency and risk of PCOS of ≥1.86 with 80% power and an alpha of 5%.

## Supplementary information

Data set 1.

## Data Availability

The primary data for this study is available from the corresponding author on direct request.
