# Supplementary material for: Genotype-independent association between vitamin D deficiency and polycystic ovarian syndrome in Lahore, Pakistan
Source: Sci Rep. 2020 Feb 10;10:2290. doi: 10.1038/s41598-020-59228-4 (PMC7010676; doi:10.1038/s41598-020-59228-4)
Supplement: Supplementary file 1 — Data set 1. [file 41598_2020_59228_MOESM1_ESM.docx]

**Genotype-independent association between vitamin D deficiency and polycystic ovarian syndrome in Lahore, Pakistan: Supplementary Material**

Nasira M. Lone^1,2*^, Saba Riaz^1,4^ , Amna Z. Eusaph^3^, Charles A. Mein^2^, Eva L.Wozniak^2^, Theodoros Xenakis^2,^ Zhenqiang Wu^5^, Sidra Younis^2,6^, David A. Jolliffe^2^ ,Kashaf Junaid^7^,Adrian R. Martineau^2^

1. Department of Microbiology and Molecular Genetics, University of the Punjab,Quaid-e-Azam Campus, Lahore 54000, Pakistan

2.Institute for Population Health Sciences, Barts and The London School of Medicine and Dentistry, Queen Mary University of London, London E1 2AB, UK

3. Lady Willingdon Hospital, Ravi Road, Walled City, Lahore 54000, Pakistan

4. Citilab and Research Center, 525-A Maulana Shaukat Ali Road, Block A Faisal Town, Lahore 54000, Pakistan

5. School of Population Health, The University of Auckland, Auckland 1142, New Zealand

6. National University of Medical Sciences, Rawalpindi 46000, Pakistan

7. Department of Clinical Laboratory Sciences, College of Applied Medical Sciences, Jouf University, Sakakah, Saudi Arabia.

* To whom correspondence should be addressed at Department of Microbiology and Molecular Genetics, University of the Punjab, Quaid-e-Azam Campus, PO Box No 54590, Lahore 54000, Pakistan. Telephone: +92 3324166497. Email [nasiramunawar@gmail.com](mailto:nasiramunawar@gmail.com)

**Supplementary Table 1.** Inventoried Taqman SNP Genotyping Assays

| **Assay ID** | **Gene** | **assay ID** | **VIC/FAM allele label** |
| --- | --- | --- | --- |
| C___1915653_20 | *CYP24A1* | rs2762939 | C/G |
| C___1915659_1_ | *CYP24A1* | rs2248137 | C/G |
| C__29958084_10 | *CYP24A1* | rs6013897 | A/T |
| C__25623452_10 | *CYP27B1* | rs4646537 | G/T |
| C__25623453_10 | *CYP27B1* | rs4646536 | A/G |
| C___3133594_30 | *DBP* | rs7041 | A/C |
| C___3133604_10 | *DBP* | rs12512631 | C/T |
| C___8278879_10 | *DBP* | rs4588 | G/T |
| C__15867778_10 | *DBP* | rs2070741 | T/G |
| C__16190886_10 | *DBP* | rs2298849 | A/G |
| C__33133925_10 | *DBP* | rs16846876 | A/T |
| C___2404008_10 | *VDR* | rs731236 | A/G |
| C___2880798_10 | *VDR* | rs4334089 | A/G |
| C___2880803_10 | *VDR* | rs10783219 | A/T |
| C___2880805_10 | *VDR* | rs4516035 | C/T |
| C___2880808_10 | *VDR* | rs11568820 | C/T, |
| C___2880811_10 | *VDR* | rs7976091 | C/T |
| C___3290655_1_ | *VDR* | rs2238136 | C/T |
| C___8716062_10 | *VDR* | rs1544410 | C/T |
| C__15823836_10 | *VDR* | rs2853559 | A/G |
| C__28977635_10 | *VDR* | rs7975232 | A/C |
| C__44841138_10 | *VDR* | rs7970314 | A/G |
| C___3120982_30 | *VDR* | rs2762934 | A/G |

**Supplementary Table 2.** Custom Taqman SNP Genotyping assay

| **Assay ID** | **Gene** | **Assay ID** | **VIC/FAM** | **Assay type** | **Forward Primer Sequence** | **Reverse Primer Sequence** |
| --- | --- | --- | --- | --- | --- | --- |
| C__12060045_20 | VDR | rs2228570 | A/G | Custom design | TGGCCTGCTTGCTGTTCTTA | GGGTCAGGCAGGGAAGTG |

**Legends:**

**Supplementary Table 1.** Inventoried Taqman SNP Genotyping Assays

**Supplementary Table 2.** Custom Taqman SNP Genotyping assay
